# Association between census‐tract Social Vulnerability Index and preterm birth rates

**DOI:** 10.1002/pmf2.70142

**Published:** 2025-10-23

**Authors:** Jocelyn V. Burridge, Rebecca J. Baer, Christina D. Chambers

**Affiliations:** ^1^ Department of Pediatrics, San Diego School of Medicine University of California La Jolla California USA

## Abstract

**Importance:**

Preterm birth (PTB) is a leading cause of neonatal morbidity and mortality. Understanding how sociodemographic factors alter the risk of PTB, as quantified by the Social Vulnerability Index (SVI), can guide the development of targeted interventions aimed at reducing PTB rates in economically/socially marginalized groups with increased risk.

**Objectives:**

This study investigated the association between overall SVI and individual SVI themes on PTB (<37 weeks) and very PTB risk (<32 weeks).

**Design:**

This was a retrospective study using data from the Study of Outcomes in Mothers and Infants (SOMI) cohort, including California births from 2018 to 2021. Hospital records from the Department of Healthcare Access and Information were linked to birth certificate records. Individual‐level data were merged with the 2020 census‐tract grid and the Centers for Disease Control and Prevention SVI shapefile. SVI and individual SVI themes (socioeconomic status, household characteristics, racial and ethnic minority status, housing type, and transportation) were categorized into quintiles. Statistical analysis was performed between July 1, 2023 and July 15, 2024.

**Setting:**

Statewide analysis of PTB by census tract in California.

**Participants:**

Study cohort consisted of singleton births with no major malformations across 9129 census tracts.

**Exposures:**

The primary exposure was census‐tract SVI.

**Main outcomes and measures:**

Study outcomes were PTB (<37 weeks’ gestation) and very PTB (<32 weeks). Mixed‐effect logistic regression models adjusted for spatial dependencies and relevant confounders.

**Results:**

This cross‐sectional study included 1,029,639 singleton births (PTB: 71,591[7.0%]) in California from January 1, 2018 to December 31, 2021. Significant associations were found between PTB and successively higher SVI quintiles compared to Very Low SVI quintile, adjusting for confounders. The highest risk was in the Very High SVI quintile (adjusted odds ratio [aOR], 1.34; 95% confidence interval [CI], [1.30, 1.38]). Increased risks were also seen for very PTB and each SVI theme.

**Conclusion and relevance:**

In this cross‐sectional study of singleton births in California, there was a significant association between higher SVI and PTB at the census‐tract level. This finding highlighted the potential for SVI and SVI themes to be used as a tool to assess the risk of PTB and guide intervention design in high‐SVI areas.

## 1 INTRODUCTION

Preterm birth (PTB), defined as delivery before 37 weeks of gestation, is a leading cause of neonatal morbidity and mortality worldwide [1]. In the United States, the complexities surrounding PTB are multifactorial, with socioeconomic factors playing a critical role [2]. Existing literature highlights the role neighborhood conditions and community health factors play in health outcomes, showing substantial heterogeneity between geographic regions [3]. Existing studies often focus on maternal characteristics, yet sociodemographic factors such as financial status, household characteristics, racial and ethnic status, and educational attainment have been shown to influence maternal and child health, including PTB rates [4, 5, 6, 7, 8]. While these factors can identify individual risk for adverse maternal and infant health outcomes, limited access to individual‐level data poses challenges in using these metrics effectively for risk prevention, especially in areas with fewer resources.

Identifying which social determinants pose the greatest risk for adverse obstetrical outcomes remains challenging, as community factors such as crime, poverty, housing, and demographics interact to increase the vulnerability of women to adverse pregnancy outcomes [9]. These risk factors do not live in isolation, but are shaped by long‐standing systemic inequities rooted and historical policies [10, 11, 12]. Examples include racial and economic spatial polarization, which have been shown to significantly influence health outcomes, including birth outcomes [13]. To account for these complexities, the Social Vulnerability Index (SVI) has emerged as a valuable tool for assessing community‐level risks. Developed by the US Centers for Disease Control and Prevention (CDC), the SVI quantifies the social vulnerability of communities to external stresses on human health based on census tract of residence. It evaluates 16 social factors across four domains: socioeconomic status, household characteristics, racial and ethnic minority status, and housing type and transportation [14].

The SVI offers a composite measure of risk that captures the interplay of multiple risk factors within communities. Historical practices such as residential segregation, redlining, and inequitable access to education and employment opportunities have contributed to the spatial polarization observed in these SVI domains, including socioeconomic status, racial and ethnic demographics, and housing and transportation access [11, 15, 16]. These inequities have resulted in concentrated vulnerabilities within certain census tracts, underscoring the importance of assessing how risk factors for PTB may interact and compound spatially [10].

SVI has demonstrated efficacy in several public health contexts, including the design of targeted interventions for communities with a higher risk of teen pregnancy, emergency medical service incidents, and certain medical risks [17, 18, 19, 20]. While preliminary data show a potential association between SVI and increased PTB risk, limited sample size and census‐tract representation lower the generalizability of results to broader geographic regions [17, 18, 19, 20, 21]. In addition, many previous studies in this area have not accounted for spatial dependencies within the data, which could lead to biased results if neighboring census tracts with shared environmental or social factors are not adequately modeled [17, 18, 19, 20, 21]. Because PTB often reflects shared neighborhood conditions rather than isolated individual risks, outcomes may cluster geographically. Standard regression approaches assume independence across units, which can obscure the contribution of contextual vulnerabilities. To address this, we applied spatial analytic methods to detect and adjust for geographic autocorrelation, ensuring that observed associations between census‐tract social vulnerability and PTB capture actual community‐level risk rather than artifacts introduced by clustering of nearby census tracts.

This study used a multilevel analysis to examine whether the SVI of a birthing person's residential census tract, independent of their individual‐level maternal characteristics, influences the risk of overall and very PTB.

## 2 METHODS

### 2.1 Study design and population

This cross‐sectional study utilized a population‐based sample of all births in California between January 1, 2018 and December 31, 2021. Data for this study were obtained from the Study of Outcomes in Mothers and Infants (SOMI), a retrospective birth cohort comprised of linked health records and vital statistics from pregnancies maintained at UC San Diego.

Hospitalization records in this cohort were retrieved from the Department of Healthcare Access and Information (HCAI) and linked to vital statistic records, from which infant birth records and maternal demographic information were obtained [22, 23]. Individual‐level residential census tract was merged with the 2020 census‐tract grid and the 2020 SVI shapefile from the CDC [14]. The study sample was restricted to singleton live births with no major malformations delivered between 2018 and 2021. The starting sample included 1,373,565 persons available in the SOMI pregnancy registry fitting these criteria. The final sample included 1,029,639 participants after further restricting for participant‐reported address in one of 9129 California census tracts (343,926 excluded).

The study was approved by the University of California San Diego Human Research Protection Program and the Committee for the Protection of Human Subjects for the State of California.

This study adhered to the STROBE (Strengthening the Reporting of Observational Studies in Epidemiology) guidelines for cross‐sectional studies. A completed STROBE checklist has been provided to ensure transparency in reporting (see the Supporting Information).

### 2.2 Primary analysis exposure

The primary exposure in this study was SVI [7, 8]. SVI measures were available at a census‐tract level and represented as percentiles derived from combined indicators across each individual census tract.

The overall SVI score encompassed the following: (1) Socioeconomic status: percentage living below 150% poverty, unemployment rate, housing cost burden, percentage with no high school diploma, and percentage with no health insurance. (2) Household characteristics: percentage of persons over 65 and below 17 years of age, disability rates, single‐parent household rates, and overall English language proficiency. (3) Racial and ethnic minority status: percentage of persons identifying as a minority group. (4) Housing type and transportation: percentages of multiunit structures, mobile homes, crowding measures, persons without vehicles, and persons living in group quarters. The SVI score is standardized from 0 to 100 nationally (0, low; 100, high).

SVI measures were divided into quintiles: Very Low (0–20), Low (20–40), Moderate (40–60), High (60–80), and Very High (80–100) in line with current research [20, 24].

### 2.3 Outcomes

The primary outcome was PTB (gestational age <37 weeks). The secondary outcome was very PTB (<32 weeks). Gestational age, reported as the best obstetric estimate, was obtained from birth certificate records.

### 2.4 Covariates

Maternal variables were included based on prior literature of PTB risk factors [25, 26, 27]. These included maternal age (<18, 18–34, >34 years), insurance status (public, private, self‐pay, and other), maternal education (<12th grade, high school and greater, unknown), body mass index (BMI <18.5, 18.5–24.9, 25.0–29.9, ≥30, missing), nulliparity (binary variable), hypertension (binary variable), pre‐existing diabetes (binary variable), gestational diabetes (binary variable), smoking during pregnancy (binary variable), and rural–urban classification (Urban Influence Codes [UICs], categorical). Race and ethnicity (American Indian or Alaska Native, Asian or Pacific Islander, Hispanic, non‐Hispanic Black, non‐Hispanic White, and >1 race or ethnicity) information was collected via birth certificates. Race/ethnicity information was included based on prior associations with the outcome of interest. However, this variable was excluded as a covariate in the final model due to concerns of overadjusting based on racial components included in SVI measures [16]. A sensitivity analysis was conducted including race/ethnicity, and results were unchanged.

### 2.5 Statistical analysis

#### 2.5.1 Descriptive statistics

Descriptive statistics were reported using counts and percentages for individual pregnancy/birth characteristics by SVI quintile. Pregnancy and birth characteristics included PTB rates (including premature rupture of the membranes [PROMs], spontaneous labor with intact membranes, and provider‐initiated birth rates [i.e., labor induction]), gestational weight, delivery characteristics, neonatal intensive care unit (NICU) admission rates, and infant characteristics.

### 2.6 Spatial analysis of PTB

Spatial analysis was incorporated to account for the possibility that PTB risk is not randomly distributed across census tracts. Birthing people residing in adjacent communities may be exposed to similar structural factors, such as environmental hazards, healthcare access, or socioeconomic disadvantage. If these spatial dependencies are ignored, regression models can underestimate standard errors and inflate the apparent precision of effect estimates, limiting their validity for public health planning. To evaluate this, we analyzed the spatial autocorrelation of regression residuals using the Global Moran's *I* statistic after fitting a baseline logistic regression model of SVI quintiles and PTB. A spatial weights matrix was constructed (excluding isolated census tracts); tracts with zero totals were assigned a value of 2, and the number of nearest neighbors was set to six based on Akaike Information Criterion (AIC) [28]. Significant clustering was observed, supporting the need for models that account for geographic dependence. We therefore incorporated a random intercept for census tract in mixed‐effects logistic regression models, which allows births within the same tract (or neighboring tracts) to be correlated. Global Moran's *I* was reapplied on the final models to confirm that residual spatial dependence was minimized. This adjustment strengthens inference by distinguishing contextual effects of social vulnerability from spatial artifacts, producing results that are more reliable for guiding geographically targeted maternal and child health interventions.

### 2.7 Regression analysis

Based on results from spatial analysis, we examined the association of overall SVI quintiles and PTB using mixed‐effects binary logistic regression for any PTB and mixed‐effects multinomial logistic regression for PTB categories, adjusting for covariates. A random intercept was included in all models to account for census‐tract‐level clustering, reflecting the nested structure of individual births within census tracts.

Models were run separately for each SVI theme and for overall SVI quintiles, with the lowest SVI quintile serving as the reference group. Adjusted odds ratios (aORs) were reported with 95% confidence intervals (CIs) and a significance level of *p* < 0.05.

All statistical analyses were conducted using R (version 4.3.2).

## 3 RESULTS

The analytic cohort included 1,029,639 singleton live births with no major birth defects, of which 71,591 (7.0%) were PTB. The distribution of participants across SVI quintiles was as follows: Very Low (141,353; 13.7%), Low (169,957; 16.5%), Moderate (191,957; 18.6%), High (236,778; 22.8%), and Very High (289,594; 27.9%). PTB rates were lowest in the Very Low SVI quintile (5.9%) and highest in the Very High SVI quintile (7.8%). Several maternal and infant characteristics were more prevalent within higher SVI quintiles, such as Hispanic race (17% for Very Low vs. 72% for Very High), Black race (1.8% for Very Low vs. 6.8% for Very High), maternal age <18 (0.1% for Very Low vs. 1.6% for Very High), public insurance payer (12% for Very Low vs. 70% for Very High), <12th grade education (1.7% for Very Low vs. 35% for Very High), and obese BMI (14% for Very Low vs. 35% for Very High). Full maternal and infant characteristics can be found in Tables 1 and 2.

**TABLE 1:** Individual maternal characteristics by quintile of SVI.

	SVI quintile (*N* = 1,029,639)				
Characteristic	Very Low (*n* (%))	Low (*n* (%))	Moderate (*n* (%))	High (*n* (%))	Very High (*n* (%))
All participants	141,353	169,957	191,957	236,778	289,594
All PTBs	8267 (5.8)	10,582 (6.2)	12,788 (6.7)	17,265 (7.3)	22,689 (7.8)
Maternal race and ethnicity					
White	69,437 (49)	68,515 (40)	59,793 (31)	43,461 (18)	28,295 (9.8)
Hispanic	23,498 (17)	46,185 (27)	77,230 (40)	141,506 (60)	208,126 (72)
Black	2610 (1.8)	5045 (3.0)	8322 (4.3)	11,462 (4.8)	19,811 (6.8)
American Indian	234 (0.2)	429 (0.3)	706 (0.4)	864 (0.4)	892 (0.3)
Hawaiian/Pacific islander	308 (0.2)	506 (0.3)	794 (0.4)	934 (0.4)	917 (0.3)
Asian	32,610 (23)	34,893 (21)	30,473 (16)	25,685 (11)	19,742 (6.8)
Two or more races	4307 (3.0)	5099 (3.0)	5332 (2.8)	4797 (2.0)	4095 (1.4)
Other/unknown	8349 (5.9)	9285 (5.5)	8867 (4.6)	8069 (3.4)	7716 (2.7)
Maternal age					
<18	209 (0.1)	585 (0.3)	1150 (0.6)	2447 (1.0)	4588 (1.6)
18–34	86,644 (61)	116,473 (69)	142,368 (74)	186,512 (79)	234,383 (81)
>34	54,500 (39)	52,899 (31)	47,999 (25)	47,819 (20)	50,623 (17)
Maternal insurance					
Private payer	117,162 (83)	122,638 (72)	112,837 (59)	100,894 (43)	77,995 (27)
Public payer	16,497 (12)	38,909 (23)	70,147 (37)	126,890 (54)	203,344 (70)
Self‐pay	3714 (2.6)	3817 (2.2)	4061 (2.1)	4953 (2.1)	4444 (1.5)
Other/unknown	3980 (2.8)	4593 (2.7)	4472 (2.3)	4041 (1.7)	3811 (1.3)
Maternal educational attainment					
Less than grade 12	2137 (1.7)	6303 (4.4)	13,822 (9.3)	32,489 (20)	66,835 (35)
Grade 12 or higher	117,203 (91)	126,094 (87)	122,698 (82)	120,759 (73)	109,135 (57)
Unknown	9982 (7.7)	12,108 (8.4)	12,470 (8.4)	13,075 (7.9)	14,476 (7.6)
Maternal BMI category					
Underweight	5735 (4.1)	5982 (3.5)	6098 (3.2)	6506 (2.7)	6892 (2.4)
Average	82,433 (58)	85,601 (50)	81,240 (42)	84,182 (36)	90,512 (31)
Overweight	32,120 (23)	42,844 (25)	52,133 (27)	67,764 (29)	84,958 (29)
Obese	19,360 (14)	33,073 (19)	48,740 (25)	73,673 (31)	100,128 (35)
Unknown	1705 (1.2)	2457 (1.4)	3306 (1.7)	4653 (2.0)	7104 (2.5)
Parity					
Nulliparous	62,105 (44)	75,353 (44)	80,032 (42)	88,868 (38)	96,549 (33)
Multiparous	79,196 (56)	94,522 (56)	111,362 (58)	147,778 (62)	192,861 (67)
Pregnancy characteristics					
Infection complicating pregnancy	8872 (6.3)	12,320 (7.2)	17,271 (9.0)	24,618 (10)	34,812 (12)
Maternal anemia	14,656 (10)	19,354 (11)	22,856 (12)	27,479 (12)	34,769 (12)
Very short birth interval	1663 (1.2)	2716 (1.6)	3890 (2.0)	6510 (2.7)	9647 (3.3)
Short birth interval	30,445 (22)	32,415 (19)	34,517 (18)	41,306 (17)	49,659 (17)
Long birth interval	10,763 (7.6)	17,225 (10)	24,604 (13)	37,841 (16)	53,662 (19)
Previous C‐section	21,582 (15)	25,672 (15)	30,451 (16)	41,451 (18)	54,438 (19)
Previous preterm birth	1807 (1.3)	2057 (1.2)	2345 (1.2)	2716 (1.1)	3366 (1.2)
Substance use					
Any smoking during pregnancy	1394 (1.0)	2865 (1.7)	4698 (2.5)	5866 (2.5)	7346 (2.5)
Any cannabis use	741 (0.5)	1557 (0.9)	2738 (1.4)	3842 (1.6)	5452 (1.9)
Substance use disorder	1088 (0.8)	2246 (1.3)	3662 (1.9)	5037 (2.1)	6895 (2.4)
Alcohol abuse	279 (0.2)	343 (0.2)	437 (0.2)	492 (0.2)	619 (0.2)
Drug use complicating pregnancy	1088 (0.8)	2246 (1.3)	3662 (1.9)	5037 (2.1)	6895 (2.4)
Maternal diabetes					
Pre‐existing diabetes	2173 (1.5)	3129 (1.8)	4069 (2.1)	5556 (2.3)	7591 (2.6)
Gestational diabetes	12,313 (8.7)	15,218 (9.0)	17,516 (9.1)	21,197 (9.0)	24,073 (8.3)
Any diabetes	14,486 (10)	18,347 (11)	21,585 (11)	26,753 (11)	31,664 (11)
Hypertension (HTN) disorder	16,697 (12)	21,220 (12)	24,056 (13)	27,957 (12)	33,149 (11)
Any pre‐exist HTN	3296 (2.3)	4481 (2.6)	5318 (2.8)	6537 (2.8)	7996 (2.8)
Pre‐exist and preeclampsia	1194 (0.8)	1694 (1.0)	2072 (1.1)	2604 (1.1)	3188 (1.1)
Pre‐exist w/o preeclampsia	2102 (1.5)	2787 (1.6)	3246 (1.7)	3933 (1.7)	4808 (1.7)
Any gest HTN	12,049 (8.5)	15,143 (8.9)	16,821 (8.8)	19,195 (8.1)	22,361 (7.7)
Gest HTN and preeclampsia	5659 (4.0)	7326 (4.3)	8352 (4.4)	9752 (4.1)	11,907 (4.1)
Gest HTN w/o preeclampsia	6390 (4.5)	7817 (4.6)	8469 (4.4)	9443 (4.0)	10,454 (3.6)
Unspecified HTN	1352 (1.0)	1596 (0.9)	1917 (1.0)	2225 (0.9)	2792 (1.0)
Maternal birthplace					
The United States	97,061 (69)	115,213 (68)	130,874 (68)	153,833 (65)	174,642 (60)
Mexico	3997 (2.8)	10,155 (6.0)	19,297 (10)	41,991 (18)	68,876 (24)
Other	40,295 (29)	44,589 (26)	41,346 (22)	40,954 (17)	46,076 (16)
Women, infants, and children (WIC) program participation					
No	96,668 (91)	104,331 (81)	97,810 (67)	88,768 (49)	70,427 (32)
Yes	9303 (8.7)	23,921 (19)	47,057 (32)	91,940 (51)	151,374 (68)
Unknown	679 (0.6)	911 (0.7)	1008 (0.7)	1186 (0.7)	1217 (0.5)
Mental health					
Maternal anxiety	8316 (5.9)	9587 (5.6)	10,723 (5.6)	11,023 (4.7)	10,970 (3.8)
Maternal depression	5555 (3.9)	6768 (4.0)	7647 (4.0)	7774 (3.3)	7569 (2.6)
Urban/rural classification					
Large central metro	98,243 (70)	114,115 (67)	122,258 (64)	149,004 (63)	181,310 (63)
Large fringe metro	24,452 (17)	22,591 (13)	22,658 (12)	25,850 (11)	21,682 (7.5)
Medium metro	17,720 (13)	30,349 (18)	41,074 (21)	56,932 (24)	83,799 (29)
Small metro or nonmetro	937 (0.7)	2890 (1.7)	5519 (2.9)	4966 (2.1)	2754 (1.0)
Unknown	1 (<0.1)	12 (<0.1)	8 (<0.1)	26 (<0.1)	49 (<0.1)

Abbreviations: BMI, body mass index; PTB, preterm birth; SVI, Social Vulnerability Index.

**TABLE 2:** Individual‐level infant characteristics by quintile of SVI.

	SVI quintile (*N* = 1,029,639)				
Characteristic	Very Low (*n* (%))	Low (*n* (%))	Moderate (*n* (%))	High (*n* (%))	Very High (*n* (%))
All participants	141,353	169,957	191,957	236,778	289,594
All PTBs	8267 (5.8%)	10,582 (6.2%)	12,788 (6.7%)	17,265 (7.3%)	22,689 (7.8%)
Moderate preterm (32–36 weeks)	7425 (5.3%)	9435 (5.6%)	11,187 (5.8%)	15,058 (6.4%)	19,671 (6.8%)
Spontaneous	1722 (1.2%)	2243 (1.3%)	2740 (1.4%)	3776 (1.6%)	5172 (1.8%)
Induced	1655 (1.2%)	2020 (1.2%)	2316 (1.2%)	2892 (1.2%)	3424 (1.2%)
PROM	2178 (1.5%)	2461 (1.4%)	2739 (1.4%)	3266 (1.4%)	3786 (1.3%)
Very preterm (<32 weeks)	842 (0.6%)	1147 (0.7%)	1601 (0.8%)	2207 (0.9%)	3018 (1.0%)
Spontaneous	188 (0.1%)	267 (0.2%)	403 (0.2%)	508 (0.2%)	686 (0.2%)
Induced	131 (<0.1%)	196 (0.1%)	241 (0.1%)	310 (0.1%)	397 (0.1%)
PROM	237 (0.2%)	313 (0.2%)	395 (0.2%)	547 (0.2%)	704 (0.2%)
Gestational weight					
Small for gestational age	11,200 (7.9%)	14,022 (8.3%)	16,116 (8.4%)	20,497 (8.7%)	26,171 (9.0%)
Average for gestational age	116,995 (83%)	139,925 (82%)	156,866 (82%)	193,464 (82%)	235,520 (81%)
Large for gestational age	13,104 (9.3%)	15,916 (9.4%)	18,398 (9.6%)	22,602 (9.5%)	27,606 (9.5%)
Unknown	73 (<0.1%)	115 (<0.1%)	160 (<0.1%)	235 (<0.1%)	326 (0.1%)
Birth characteristics					
C‐section delivery	35,150 (25%)	41,454 (24%)	45,227 (24%)	54,031 (23%)	62,421 (22%)
Fetal abruption	1315 (0.9%)	1493 (0.9%)	1803 (0.9%)	2072 (0.9%)	2462 (0.9%)
NICU type					
Intermediate NICU	6919 (4.9%)	7120 (4.2%)	8099 (4.2%)	9861 (4.2%)	15,993 (5.5%)
Community NICU	46,275 (33%)	59,890 (35%)	68,732 (36%)	98,653 (42%)	129,574 (45%)
Regional NICU	15,880 (11%)	15,991 (9.4%)	14,571 (7.6%)	10,739 (4.5%)	11,839 (4.1%)
Infant sex					
Male	71,529 (51%)	86,092 (51%)	96,790 (51%)	119,330 (50%)	146,269 (51%)
Female	69,824 (49%)	83,863 (49%)	94,724 (49%)	117,446 (50%)	143,323 (49%)
Unknown	0 (0%)	2 (<0.1%)	2 (<0.1%)	1 (<0.1%)	1 (<0.1%)

Abbreviations: NICU, neonatal intensive care unit; PROM, premature rupture of the membrane; PTB, preterm birth; SVI, Social Vulnerability Index.

### 3.1 PTB spatial analysis

The Global Moran's *I* result (*I* = 0.2, standard deviate = 33.443, *p* < 2.2e−16) in the initial binary logistic regression model indicated a moderate and significant spatial autocorrelation of residuals, implying a nonrandom spatial pattern of PTB. To address this, a random intercept for census tract was added to the regression model to account for spatial clustering. After incorporating the random intercept, the residuals of the multilevel model showed minimal spatial autocorrelation, with a Global Moran's *I* of 0.004 (standard deviate = 0.62, *p* = 0.2684), indicating that most of the spatial dependence had been accounted for by the mixed‐effects model.

### 3.2 Overall PTB and SVI association

After adjusting for potential confounders, the association between SVI and any PTB showed a dose–response relationship. Compared to mothers in the Very Low category, those in the Low SVI category had a slightly increased likelihood of PTB (aOR, 1.04; 95% CI, [1.01–1.07]), while those in the Moderate SVI category had a further increased likelihood (aOR, 1.10; 95% CI, [1.07–1.14]). The trend continued with a more pronounced increase in the High SVI group (aOR, 1.25; 95% CI, [1.21–1.28]), and the highest likelihood was observed in the Very High SVI category (aOR, 1.34; 95% CI, [1.30–1.38]) (Table 3).

**TABLE 3:** Model of association between overall SVI and preterm birth.

	All preterm <37 weeks		Moderate preterm (32–36 weeks)		Very preterm (<32 weeks)	
	Unadjusted OR	Adjusted OR	Unadjusted OR	Adjusted OR	Unadjusted OR	Adjusted OR
Composite SVI						
Very Low SVI	Ref	Ref	Ref	Ref	Ref	Ref
Low SVI	1.07 (1.04, 1.10)^*^	1.04 (1.01, 1.07)^*^	1.06 (1.03, 1.09)^*^	1.03 (1.00, 1.06)	1.13 (1.03, 1.24)^*^	1.12 (1.02, 1.23)^*^
Moderate SVI	1.15 (1.12, 1.19)^*^	1.10 (1.07, 1.14)^*^	1.12 (1.09, 1.15)^*^	1.08 (1.04, 1.11)^*^	1.41 (1.29, 1.53)^*^	1.33 (1.21, 1.46)^*^
High SVI	1.27 (1.23, 1.30)^*^	1.25 (1.21, 1.28)^*^	1.23 (1.19, 1.26)^*^	1.20 (1.16, 1.24)^*^	1.57 (1.45, 1.70)^*^	1.57 (1.44, 1.72)^*^
Very High SVI	1.37 (1.33, 1.40)^*^	1.34 (1.30, 1.38)^*^	1.31 (1.28, 1.35)^*^	1.33 (1.21, 1.46)^*^	1.76 (1.63, 1.90)^*^	1.78 (1.63, 1.94)^*^

*Note*: Adjusted for individual‐level maternal age, payer, BMI, nulliparous, education level, urban/rural status, ever smoking, any hypertension, gestational diabetes, pre‐existing diabetes. Adjusted for census‐tract random effect.

Abbreviations: BMI, body mass index; OR, odds ratio; SVI, Social Vulnerability Index.

* Significant at *p* < 0.05.

In sensitivity analyses stratified by PTB subtype, dose–response associations were evident for both spontaneous and iatrogenic PTB, with a stronger gradient for spontaneous PTB (aORs ranging from 1.03 in Low SVI to 1.36 in Very High SVI) than for iatrogenic PTB (aORs ranging from 1.04 in Low SVI to 1.13 in Very High SVI) (Table 4).

**TABLE 4:** Model of association between overall SVI and preterm birth subtypes.

	Spontaneous preterm birth <37 weeks		Iatrogenic preterm birth <37 weeks	
	Unadjusted OR	Adjusted OR	Unadjusted OR	Adjusted OR
Composite SVI				
Very Low SVI	Ref	Ref	Ref	Ref
Low SVI	1.09 (1.03, 1.16)^*^	1.03 (0.96, 1.10)	1.03 (0.97, 1.10)	1.04 (0.97, 1.11)
Moderate SVI	1.22 (1.15, 1.29)^*^	1.11 (1.05, 1.19)^*^	1.06 (1.00, 1.12)	1.09 (1.02, 1.17)^*^
High SVI	1.35 (1.27, 1.42)^*^	1.25 (1.18, 1.33)^*^	1.07 (1.01, 1.14)^*^	1.17 (1.10, 1.25)^*^
Very High SVI	1.51 (1.43, 1.59)^*^	1.36 (1.28, 1.44)^*^	1.04 (0.99, 1.11)	1.13 (1.06, 1.21)^*^

*Note*: Adjusted for individual‐level maternal age, payer, BMI, nulliparous, education level, urban/rural status, ever smoking, any hypertension, gestational diabetes, pre‐existing diabetes. Adjusted for census‐tract random effect.

Abbreviations: BMI, body mass index; OR, odds ratio; SVI, Social Vulnerability Index.

* Significant at *p* < 0.05.

### 3.3 Very PTB and Overall SVI association

The patterns observed were more pronounced with very PTB. Compared to Very Low SVI, aORs indicated a progressively higher risk from Low SVI (aOR, 1.12; 95% CI, [1.02–1.23]) to Very High SVI (aOR, 1.78; 95% CI, [1.63–1.94]), alongside other SVI categories (Table 3).

### 3.4 PTB and SVI theme association

All individual SVI themes in Moderate, High, and Very High categories showed statistically significant associations with overall and very PTB rates when compared with Very Low SVI at *p* < 0.05, after adjustment for covariates (Tables 5 and 6).

**TABLE 5:** Model of association between SVI theme and any preterm birth.

	Unadjusted OR (95% CI)	Adjusted OR (95% CI)
Theme 1: Socioeconomic status		
Very Low SVI	Ref	Ref
Low SVI	1.06 (1.03, 1.09)^*^	1.03 (1.0, 1.06)
Moderate SVI	1.14 (1.11, 1.17)^*^	1.11 (1.07, 1.14)^*^
High SVI	1.24 (1.21, 1.27)^*^	1.23 (1.19, 1.26)^*^
Very High SVI	1.35 (1.32, 1.39)^*^	1.33 (1.29, 1.37)^*^
Theme 2: Household characteristics		
Very Low SVI	Ref	Ref
Low SVI	1.07 (1.04, 1.10)^*^	1.07 (1.04, 1.11)^*^
Moderate SVI	1.15 (1.12, 1.18)^*^	1.15 (1.12, 1.19)^*^
High SVI	1.23 (1.20, 1.26)^*^	1.22 (1.18, 1.25)^*^
Very High SVI	1.30 (1.27, 1.34)^*^	1.30 (1.26, 1.34)^*^
Theme 3: Racial and ethnic minority status		
Very Low SVI	Ref	Ref
Low SVI	1.10 (1.07, 1.14)^*^	1.09 (1.05, 1.22)^*^
Moderate SVI	1.19 (1.16, 1.23)^*^	1.18 (1.15, 1.22)^*^
High SVI	1.30 (1.27, 1.34)^*^	1.30 (1.26,1.34)^*^
Very High SVI	1.38 (1.35, 1.42)^*^	1.39 (1.34,1.43)^*^
Theme 4: Housing type and transportation		
Very Low SVI	Ref	Ref
Low SVI	1.09 (1.06, 1.12)^*^	1.05 (1.02, 1.09)^*^
Moderate SVI	1.14 (1.11, 1.17)^*^	1.10 (1.07, 1.13)^*^
High SVI	1.17 (1.14, 1.20)^*^	1.12 (1.09, 1.16)^*^
Very High SVI	1.23 (1.20, 1.26)^*^	1.18 (1.15, 1.22)^*^

*Note*: Adjusted for individual‐level maternal age, payer, BMI, nulliparous, education level, urban/rural status, ever smoking, any hypertension, gestational diabetes, pre‐existing diabetes. Adjusted for census‐tract random effect.

Abbreviations: BMI, body mass index; CI, confidence interval; OR, odds ratio; SVI, Social Vulnerability Index.

* Significant at *p* < 0.05

**TABLE 6:** Model of association between SVI theme and very preterm birth.

	Unadjusted OR (95% CI)	Adjusted OR (95% CI)
Theme 1: Socioeconomic status		
Very Low SVI	Ref	Ref
Low SVI	1.12 (1.02, 1.22)^*^	1.10 (1.00, 1.21)^*^
Moderate SVI	1.33 (1.22, 1.44)^*^	1.28 (1.17, 1.40)^*^
High SVI	1.53 (1.42, 1.65)^*^	1.55 (1.42, 1.69)^*^
Very High SVI	1.73 (1.61, 1.86)^*^	1.78 (1.63, 1.94)^*^
Theme 2: Household characteristics		
Very Low SVI	Ref	Ref
Low SVI	1.08 (1.00, 1.18)	1.10 (1.01, 1.21)^*^
Moderate SVI	1.23 (1.14, 1,33)^*^	1.25 (1.14, 1.36)^*^
High SVI	1.39 (1.29, 1.50)^*^	1.42 (1.31, 1.55)^*^
Very High SVI	1.51 (1.40, 1.62)^*^	1.58 (1.46, 1.73)^*^
Theme 3: Racial and ethnic minority status		
Very Low SVI	Ref	Ref
Low SVI	1.13 (1.04, 1.23)^*^	1.14 (1.03, 1.25)^*^
Moderate SVI	1.34 (1.23, 1.46)^*^	1.34 (1.22, 1.47)^*^
High SVI	1.55 (1.43, 1.68)^*^	1.53 (1.39, 1.68)^*^
Very High SVI	1.72 (1.59, 1.86)^*^	1.79 (1.64, 1.97)^*^
Theme 4: Housing type and transportation		
Very Low SVI	Ref	Ref
Low SVI	1.17 (1.08, 1.28)^*^	1.15 (1.05, 1.26)^*^
Moderate SVI	1.35 (1.25, 1.46)^*^	1.28 (1.17, 1.39)^*^
High SVI	1.37 (1.27, 1.48)^*^	1.31 (1.20, 1.43)^*^
Very High SVI	1.42 (1.32, 1.54)^*^	1.37 (1.25, 1.49)^*^

*Note*: Adjusted for individual‐level maternal age, payer, BMI, nulliparous, education level, urban/rural status, ever smoking, any hypertension, gestational diabetes, pre‐existing diabetes. Adjusted for census‐tract random effect.

Abbreviations: BMI, body mass index; CI, confidence interval; OR, odds ratio; SVI, Social Vulnerability Index.

* Significant at *p* < 0.05.

Theme 1 Socioeconomic status

For socioeconomic status, aORs showed a gradual increase in the risk of PTB with each ascending SVI category, with the highest risk seen in the Very High SVI (aOR, 1.33; 95% CI, [1.29–1.37]). For very PTB, the Very High SVI group showed a significantly increased risk (aOR, 1.78; 95% CI, [1.63–1.94]) compared to the reference group.

Theme 2 Household characteristics

Within household characteristics, both aORs suggested that SVI and PTB risk increase together, with the Very High SVI group exhibiting the highest risk increase (aOR, 1.30; 95% CI, [1.26–1.34]). Similarly, household characteristics showed a positive association with very PTB, with the highest SVI category reflecting an aOR of 1.58 (95% CI, [1.46–1.73]).

Theme 3 Racial and ethnic minority status

The racial and ethnic minority status theme also showed an upward trend in PTB risk as SVI category increased, with the highest SVI group having an aOR of 1.39 (95% CI, [1.34–1.43]), indicating a substantially higher risk of PTB. For very PTB, the highest SVI group showed the most elevated risk of very PTB (aOR, 1.79; 95% CI, [1.64–1.97]).

Theme 4 Housing type and transportation

Housing type and transportation were consistent with previous themes, with the Very High SVI group presenting an increased risk (aOR 1.18; 95% CI, [1.15–1.22]). This theme was also significantly associated with an increased risk of very PTB, with the Very High SVI group having an aOR of 1.37 (95% CI, [1.25–1.49]).

## 4 DISCUSSION

The findings of this study suggest a significant association between higher SVI scores and PTB rates at a census‐tract level in California. Each of the four SVI themes was independently associated with higher odds of PTB and very PTB, with the most substantial effects observed at the earliest gestational age cutoff.

The spatial analysis component of this study revealed significant clustering of high PTB rates in census tracts with elevated SVI scores. This pattern underscores the importance of geographically targeted interventions and highlights how PTB risk can vary substantially from one neighborhood to the next. By using census‐tract‐level data and applying spatial methods, we were able to account for similarities among neighboring communities, producing more reliable estimates of contextual risk than analyses conducted at broader geographic scales. Together, these methods show that PTB disparities follow clear geographic patterns linked to concentrated social risk. Community‐level disparities in PTB are shaped by enduring inequities in policy and resource distribution, underscoring the importance of place‐based analytic approaches and the opportunity to target interventions to the neighborhoods where they are most needed [10, 11, 15, 16].

Previous research has identified several independent social determinants of health (SDOH) as contributors to the risk of adverse pregnancy outcomes, including African American race, rural residence, education status, and low socioeconomic status [29]. More specialized spatial composite measures have also successfully used local measures of racial and economic segregation as proxies for structural racism, demonstrating an association with PTB and infant mortality [16]. The spatial clustering of SDOH provides an integrated method to better understand the combination of these independent risk factors and use geographic data as a proxy for individual‐level data. Recent studies have used this method and employed different measures, such as the Neighborhood Deprivation Index, Maternal Vulnerability Index (MVI), and SVI, to assess associations between maternal geography and PTB risk [21, 25, 30].

Among available indices, the SVI stands out for its granularity and comprehensiveness. While other indices, such as the Townsend Deprivation Index, incorporate fewer variables and operate at broader geographic scales, the SVI provides census‐tract‐level data and evaluates 16 social factors across four domains. This granularity enables a more precise analysis of how maternal geographic residence influences PTB risk [10].

By using census‐tract SVI and incorporating patient‐level data across a large geographic region, we were able to directly examine the relationship between maternal location of residence, irrespective of individual characteristics, and PTB risk. Existing studies, while supportive of our findings, often have small sample sizes and restricted participants to one delivery hospital, limiting geographic variation and reducing the generalizability of findings [21]. In addition, by including a random effect to adjust for spatial clustering, we were able to isolate the association between SVI and PTB to reflect the broader relationship. Findings from this study demonstrate the efficacy of census‐tract‐level deprivation data to identify areas of higher PTB risk, providing openings for informed interventions and improved care.

This study moves beyond confirming that social disadvantage is linked to PTB by demonstrating, at the census‐tract level across an entire state, that SVI can be used as a reliable tool to identify where inequities are most concentrated and where interventions may be most effective. Among the four SVI themes, socioeconomic status and racial/ethnic minority status showed the strongest associations with PTB, particularly very PTB, highlighting specific domains that may drive the greatest disparities. This level of geographic and thematic precision provides *actionable guidance for public health planning*: High‐SVI tracts can be prioritized for enhanced prenatal care access (e.g., home visiting, group prenatal care, community doula support) and for targeted community‐based investments. By explicitly showing that tract‐level vulnerability predicts PTB even after adjusting for individual‐level factors and spatial clustering, our study offers a *scalable, place‐based strategy* to direct limited resources to neighborhoods where interventions are most urgently needed. This approach transforms SVI from a descriptive measure of inequity into a *practical tool for public health*, supporting targeted policies and programs designed to reduce PTB disparities.

### 4.1 Limitations and strengths

This study has several limitations. Exclusion due to incomplete linkage data, multiple births, non–live births, and malformations could introduce selection bias, potentially affecting the generalizability of the findings. This study is also based on data from California, and findings may not be generalizable to other contexts. Although adjustments were made for several individual‐level confounders, other unmeasured variables, such as health‐seeking behaviors, may still influence PTB risk. Other factors associated with PTB, such as maternal autoimmune diseases, were unable to be accounted for in the analysis. Future studies incorporating detailed medical histories will allow for a more comprehensive understanding of these associations.

Additionally, this study did not differentiate between spontaneous PTB and medically indicated PTB. These two pathways to PTB are associated with distinct etiologies and risk factors. Separating them could provide deeper insights into the relationship between SVI and PTB. However, our primary goal was to assess the broad association between SVI and PTB risk, and distinguishing these subtypes was beyond the scope of this analysis. Although the study period included the COVID‐19 pandemic (2020–2021), sensitivity analyses did not indicate substantial deviations in the association between SVI and PTB during this time, suggesting that our findings remain robust despite potential pandemic‐related disruptions to healthcare access or birth outcomes.

Despite limitations, the study has notable strengths. The inclusion of over a million births strengthens the statistical power of the study and reinforces findings from previous research with limited participant numbers and demographics. Using SVI also provided comprehensive measures of community‐level risk factors, and the use of census‐tract‐level data allowed for a more precise examination of the spatial distribution of PTB risk. Additionally, the study adjusts for a wide range of individual‐level covariates and spatial clustering, minimizing the risk of confounding and providing clearer insights into the relationship between SVI and PTB.

## 5 CONCLUSION

In this cross‐sectional study of 1,029,639 live singleton births, there was a significant association between higher SVI and PTB at the census‐tract level in California. The association was strongest for very PTB and was significant across all four individual SVI themes. These results reinforce the importance of considering social determinants of health in maternal and child health research and demonstrate that health status alone does not dictate pregnancy outcomes, but that the environments in which people live also play a critical role. Beyond documenting disparities, these findings underscore the need for *place‐based interventions* that address structural and community‐level drivers of PTB risk. Future research should build on this evidence by clarifying the mechanisms linking social vulnerability and PTB and by evaluating the effectiveness of interventions tailored to communities with the greatest disadvantage. By integrating social vulnerability into both research and practice, this work can inform strategies to mitigate PTB outcomes and advance equity in maternal and infant health.

## AUTHOR CONTRIBUTIONS

**Jocelyn Burridge**: Conceptualization; methodology; software; formal analysis; writing—original draft; writing—review and editing. **Rebecca Baer**: Resources; data curation; validation; writing—review and editing. **Christina Chambers**: Supervision; writing—review and editing.

## CONFLICT OF INTEREST STATEMENT

None of the authors have a conflict of interest to disclose.

## ETHIC STATEMENT

The study was approved by the University of California San Diego Human Research Protection Program and Committee for the Protection of Human Subjects for the State of California. The authors were responsible for the design and conduct of the study; collection, management, analysis, and interpretation of the data; preparation, review, or approval of the manuscript; and decision to submit the manuscript for publication. All authors had full access to all the data in the study and take responsibility for the integrity of the data and the accuracy of the data analysis.

## Supporting information



Supporting information
